# Iron Oxide Nanoparticles with and without Cobalt Functionalization Provoke Changes in the Transcription Profile via Epigenetic Modulation of Enhancer Activity

**DOI:** 10.1021/acs.nanolett.3c01967

**Published:** 2023-07-26

**Authors:** Federica Gamberoni, Marina Borgese, Christina Pagiatakis, Ilaria Armenia, Valeria Grazù, Rosalba Gornati, Simone Serio, Roberto Papait, Giovanni Bernardini

**Affiliations:** † Department of Biotechnology and Life Sciences, University of Insubria, via J.H. Dunant 3, 21100 Varese, Italy; ‡ Department of Medicine and Surgery, University of Insubria, via Guicciardini 9, 21100 Varese, Italy; § IRCCS Humanitas Research Hospital, via Manzoni 56, 20089 Rozzano, Milan, Italy; ∥ BioNanoSurf Group, Instituto de Nanociencia y Materiales de Aragón (INMA, CSIC-UNIZAR), Edificio I + D, 50018 Zaragoza, Spain; ⊥ Department of Biomedical Sciences, Humanitas University, via Rita Levi Montalcini 4, 20072 Pieve Emanuele, MI, Italy

**Keywords:** nanoparticles, iron, cobalt, epigenetics, enhancers, promoters, nanotoxicity, ChIP-seq, RNA-seq, histone modifications

## Abstract

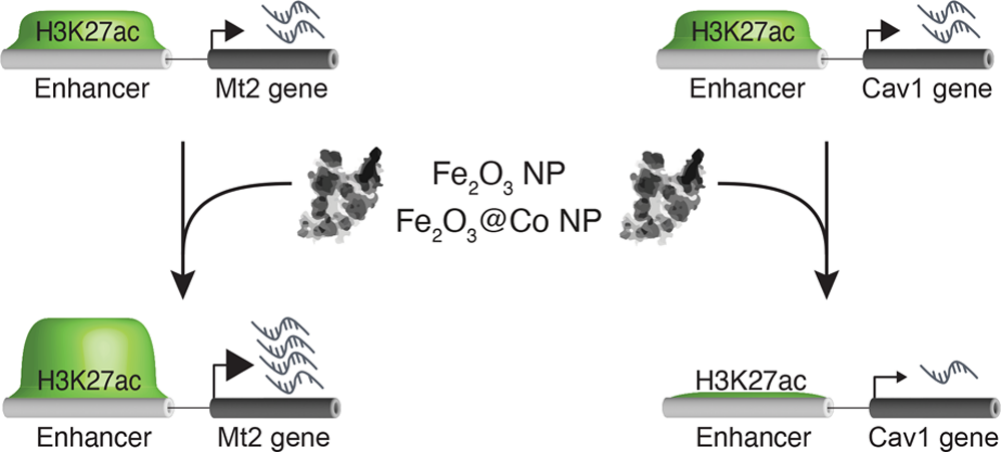

Despite the progress in the field of nanotoxicology, much about the cellular mechanisms that mediate the adverse effects of nanoparticles (NPs) and, in particular, the possible role of epigenetics in nanotoxicity, remains to be clarified. Therefore, we studied the changes occurring in the genome-wide distribution of H3K27ac, H3K4me1, H3K9me2, and H3K27me3 histone modifications and compared them with the transcriptome after exposing NIH3T3 cells to iron-based magnetic NPs (i.e., Fe_2_O_3_ and Fe_2_O_3_@Co NPs). We found that the transcription response is mainly due to changes in the genomic distribution of H3K27ac that can modulate the activity of enhancers. We propose that alteration of the epigenetic landscape is a key mechanism in defining the gene expression program changes resulting in nanotoxicity. With this approach, it is possible to construct a data set of genomic regions that could be useful for defining toxicity in a manner that is more comprehensive than what is possible with the present toxicology assays.

Iron-based nanoparticles (NPs) are widely used in many areas of everyday life, including environmental remediation,^1^ magnetic resonance imaging,^2^ cancer treatment,^3^ food supplementation,^4^ and targeting of antibiotics,^5,6^ enzymes,^7^ and other drugs. Therefore, it is critical to assess their potential toxicity as thoroughly as possible. *In vitro*(8) and *in vivo* experiments on mammals^9,10^ and other vertebrates^11^ have shown that iron-based NPs are relatively toxic. Despite this, the adverse effects of these types of NPs were reported, including impairment of cell viability, oxidative stress in the lung, inflammatory reactions, and blood coagulation alterations.^12^

The current toxicological assays are limiting because they do not provide a comprehensive idea of the biological effects of a substance by measuring only a few parameters, and very importantly, they fail to evaluate latent toxicity. Therefore, we need new approaches for a broader understanding of the effects of iron-based NPs on biological systems. To this end, a powerful tool is the integration of data obtained by global “omics” approaches for the study of the transcriptome, the epigenome, the proteome, and the metabolome.^13^

Chemical modifications of histone H3, such as acetylation and methylation, are an important epigenetic mechanism for the regulation of gene expression.^14^ The use of omics approaches to study the epigenome [e.g., chromatin immunoprecipitation coupled with massively parallel sequencing (ChIP-seq)] and the transcriptome [e.g., RNA sequencing (RNA-seq)] has revealed that these epigenetic marks determine the transcription programs underlying cell differentiation and the maintenance of tissue homeostasis in adults^15,16^ and how alteration of their genomic distribution can cause transcriptional changes leading to several human diseases (e.g., cancer, diabetes, neurological and neurodegenerative diseases, and cardiovascular disorders).^17−19^ Histone H3 markers are also involved in mediating the effects of several environmental factors (e.g., diet, exercise, and circadian rhythms) on cells.^20^

Although these findings support the idea that histone modifications could be regulated as a result of NP toxicity, and recent studies describe the alteration of the genomic distribution of some histone markers after exposure to several types of NPs (e.g., SiO_2_, TiO_2_, Au, and Ag NPs),^21,22^ the question of whether histone modifications are necessary to define the transcriptional changes inherent to nanotoxicity remains unanswered. To address this issue, we investigated, *in vitro*, whether Fe_2_O_3_ NPs and Fe_2_O_3_@Co NPs, two iron based-NPs with and without cobalt functionalization, respectively, that were produced for industrial application by the Europe HOTZYMES consortium (https://www.hotzymes.eu), and the ions that comprise them, namely, iron and cobalt, lead to modifications of the epigenetic landscape responsible for the gene expression changes causing nanotoxicity. To this end, we have generated genome-wide maps of four key histone H3 modifications (H3K27ac and H3K4me1, two histone marks that promote transcription activation, and H3K9me2 and H3K27me3, which are associated with transcription repression) of NIH3T3 cells (a mouse fibroblast cell line) exposed to either Fe_2_O_3_ NPs or Fe_2_O_3_@Co NPs, and furthermore, we have correlated the changes in the histone landscape to those in gene expression. Fe_2_O_3_ NPs are quasi-spherical γ-Fe_2_O_3_ NPs of 12 nm, synthesized as reported previously.^23^ These NPs were further functionalized with NTA-Co (Fe_2_O_3_@Co NPs) using the classic carbodiimide chemistry of EDC and s-NHS. To discern the effects of the two NPs from that of iron, a component of both NPs, and cobalt, present only in Fe_2_O_3_@Co NPs, we also assessed the effects of FeSO_4_ and CoCl_2_ (Figure 1A). To this end, we carried out ChIP-seq on NIH3T3 cells exposed to 6.25 μg/mL Fe_2_O_3_ NPs and Fe_2_O_3_@Co NPs, FeSO_4_, or CoCl_2_ for 48 h. All four treatments partially affected cell viability as determined by measuring their ATP content (Figure S1A).

**Figure 1:**
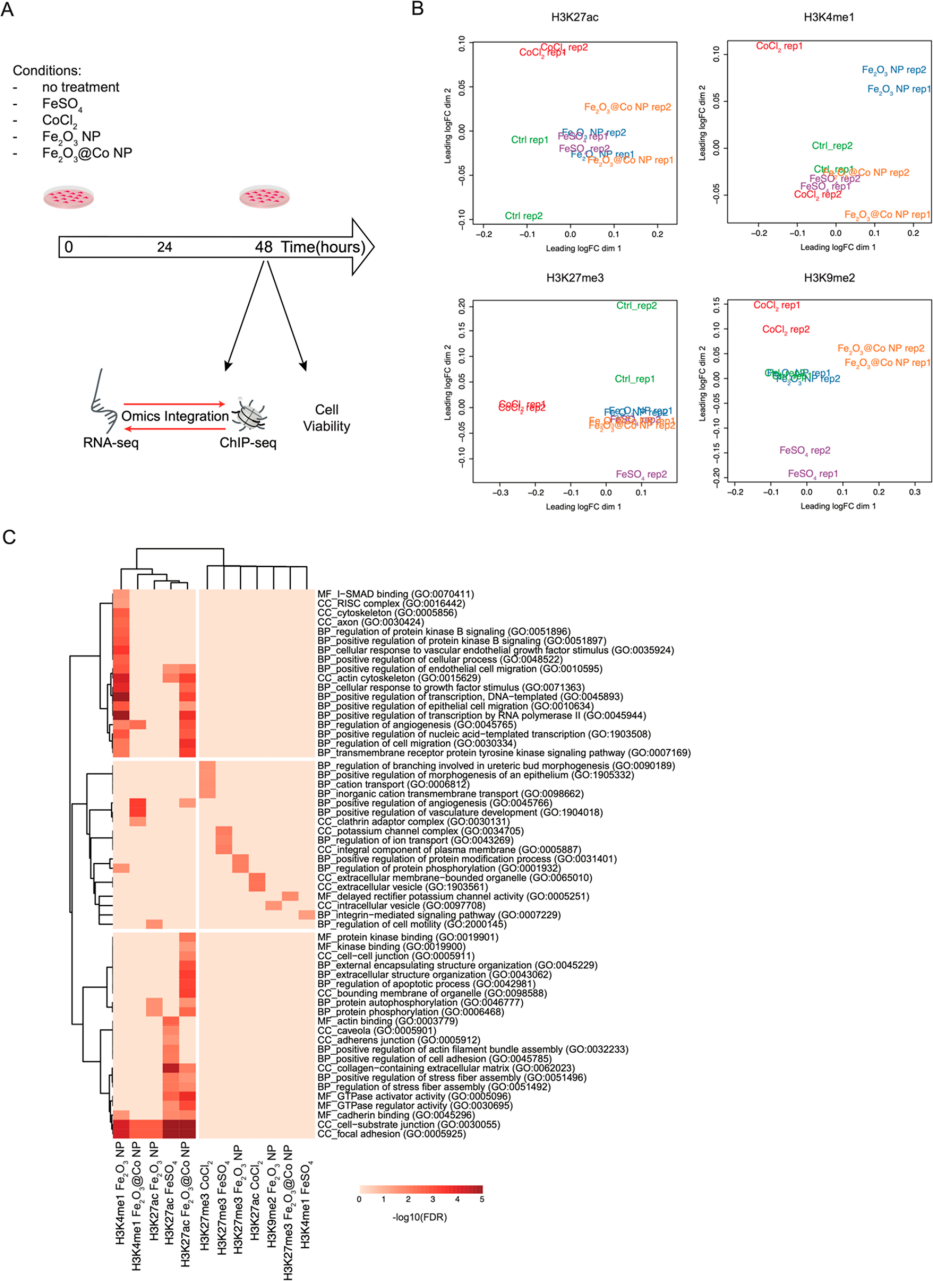
ChIP-seq analysis of H3K27ac, H3K4me1, H3K9m2, and H3K27me3 in NIH3T3 cells exposed to 6.25 μg/mL Fe_2_O_3_ nanoparticles (NPs), Fe_2_O_3_@Co NPs, FeSO_4_, or CoCl_2_ for 48 h. (A) Schematic of the experimental workflow. (B) Multidimensional scaling (MDS) plots of H3K27ac, H3K4me1 H3K9me2, and H3K27m3 to visualize the similarities based on their genomic distribution in NIH3T3 cells exposed to Fe_2_O_3_ NPs, Fe_2_O_3_@Co NPs, FeSO_4_, or CoCl_2_. (C) Heat map of the top 10 gene ontology terms [false discovery rate (FDR) < 0.05] for molecular function (MF), biological processes (BP), and cellular component (CC) for the two closest genes mapping near differential peaks for each histone modification for each treatment.

Correlation analysis of genomic regions enriched for each individual histone modification (Figure 1B) and the identification of the differential peaks (DPs), i.e., genomic regions exhibiting a significant [false discovery rate (FDR) < 0.1; |log 2FC| > 0.3] increase or decrease in the deposition of a histone modification, have revealed that each treatment had a different impact on the genomic distribution of the marks studied. Fe_2_O_3_@Co NPs and FeSO_4_ had significant effects on the genomic distribution of H3K27ac (6456 total DPs with Fe_2_O_3_@Co NPs4 and 2697 total DPs with FeSO_4_), whereas Fe_2_O_3_ NPs altered the deposition of H3K4me1 (3952 total DPs); CoCl_2_ affected H3K27me3 (15 847 total DPs) (Figure S1C and Data set S1). This indicates that Fe_2_O_3_ NPs and Fe_2_O_3_@Co NPs, as well as their corresponding iron and cobalt ions, could distinctly modify the epigenetic landscape, controlling the redistribution of histone H3 marks.

To gain further insight into the biological role of the genomic regions found with an altered distribution of histone modifications, we have performed gene ontology (GO) analysis for molecular function, biological processes, and cellular component of nearby genes. As shown in Figure 1C, there was a significant enrichment in GO terms linked with toxicity (e.g., cellular response to growth factors, regulation of angiogenesis, regulation of cell migration, adherens junctions, and regulation of apoptotic processes) for genomic regions undergoing altered H3K27ac deposition upon exposure to both the NPs and FeSO_4_, as well as for genomic regions with redistributed H3K4me1 after exposure to the NPs. Conversely, only a small number of GO terms were correlated with the differential peaks of H3K27me3 and H3K9me2. To verify whether histone mark redistribution contributed to nanotoxicity via the activation of “harmful” gene transcription programs, we have correlated ChIP-seq data with global transcriptional changes. To this end, we have performed RNA sequencing on polyadenylated RNA purified from the same corresponding samples used for ChIP-seq. Our analysis has revealed that each treatment induced a different number of differentially expressed genes (DEGs; FDR < 0.1). FeSO_4_ caused the strongest effect with 216 DEGs, whereas CoCl_2_ had 97 DEGs, Fe_2_O_3_ NPs 143 DEGs, and Fe_2_O_3_@Co 81 DEGs (Figure 2A).

**Figure 2:**
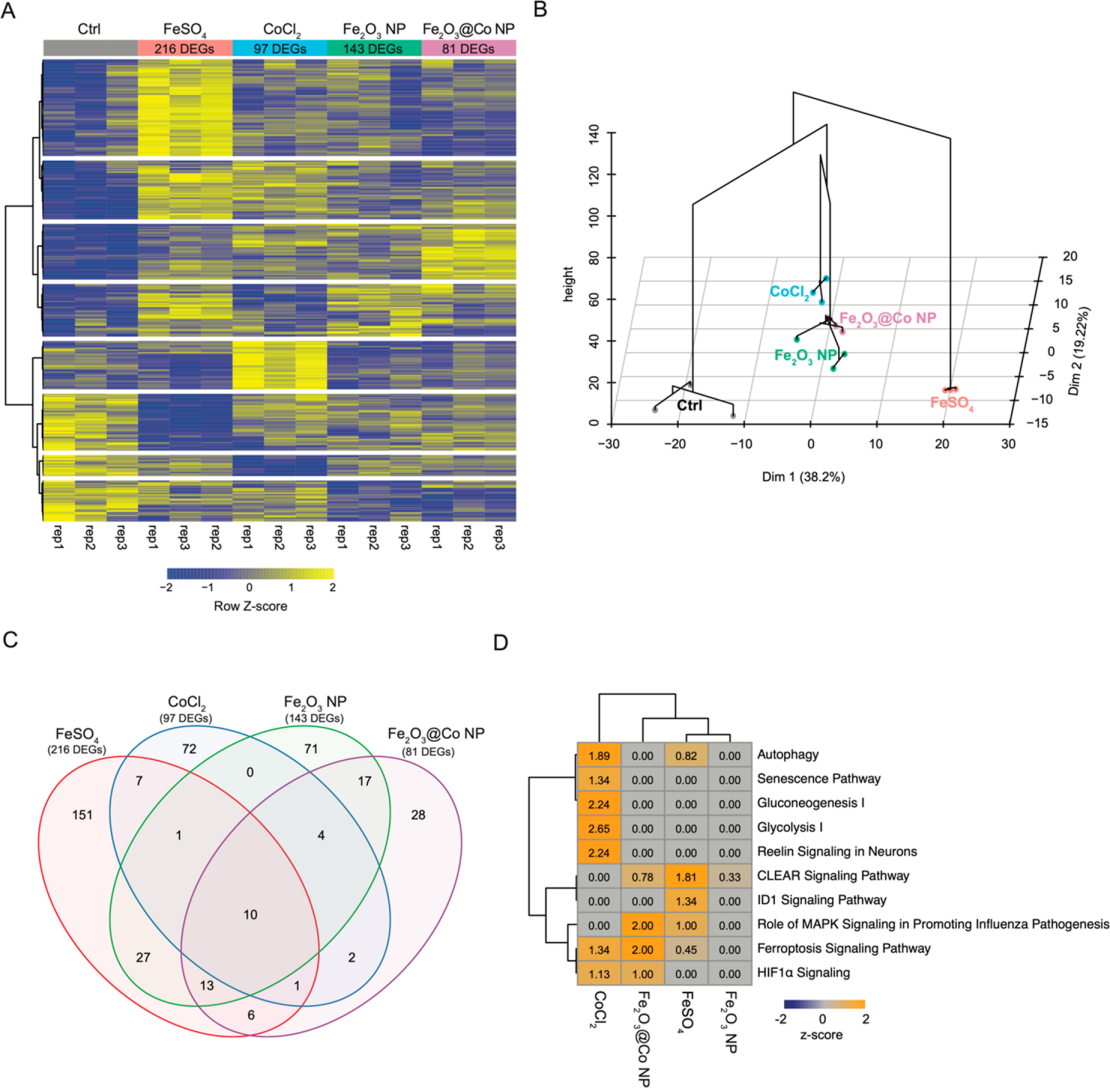
RNA-seq analysis of NIH3T3 cells exposed to 6.25 μg/mL Fe_2_O_3_ NPs, Fe_2_O_3_@Co NPs, FeSO_4_, or CoCl_2_ for 48 h. (A) Heat map of unsupervised hierarchical clustering of 537 differentially expressed genes (DEGs; FDR < 0.1) found in at least one treatment condition vs the nontreated control. (B) Hierarchical clustering of principal components based on transcription profiles. (C) Venn diagram of the overlap of DEGs found in each treatment vs the nontreated control. (D) Comparative ingenuity pathway analysis (IPA) of perturbed canonical pathways based on data sets of DEGs for each treatment vs the nontreated control. Pathway terms with |*z* score| values of >2 were considered significant.

Moreover, heat map and hierarchical cluster analysis have shown that different groups of genes were dysregulated (Figure 2A,B; the complete lists of the DEGs are given in Data set S2). In particular, Figure 2C highlights that only a small number of DEGs were similarly modulated under two or more experimental conditions, thus indicating that the different NPs and ions have a specific effect on gene expression.

Ingenuity pathway analysis (Figure 2D) has revealed that distinct pathways were significantly (|*z*-score| ≥ 2) modulated. Fe_2_O_3_@Co NPs altered the expression of genes linked to the activation of ferroptosis and MAPK signaling involved in the promotion of influenza pathogenesis, while CoCl_2_ exposure was associated with the activation of sugar metabolism (glycolysis I and gluconeogenesis I) and reelin signaling in neurons. Conversely, the data sets of DEGs for Fe_2_O_3_ NPs and FeSO_4_ did not show any significantly modulated pathways. These findings suggest that the toxic effects of the NPs and ions were generated through distinct gene expression programs.

We then analyzed the modulation of gene expression associated with regulatory regions, i.e., promoters and enhancers, exhibiting the redistribution of one of the four histone modifications studied. To identify regulatory regions, we took advantage of promoter-capture Hi-C (PCHi-C) data obtained from the 3T3-L1 preadipocyte cell line.^24^ This method is based on the identification of regulatory regions physically interacting with promoters and allows more accurate annotations than those based only on genomic distance. We have found that the transcriptional changes resulting from the two NPs were dependent on the redistribution of H3K27ac and H3K4me1, whereas those evoked by FeSO_4_ were dependent on only an altered distribution of H3K27ac. In contrast, there was no significant link between the modulation of gene expression and altered histone modification profiles in cells exposed to CoCl_2_. It is noteworthy that the two repressive histone modifications studied (i.e., H3K27me3 and H3K9me2) were not involved in defining any of the transcriptional changes observed (Figure 3A). This was also supported by analysis of changes in H3K9me2 in K9-dimethyl domains (KDDs), large heterochromatic domains playing a key role in the repression of transcription. We have found that H3K9me2 had a weak impact on the genomic dimension of KDDs (Figure S3B) and that the KDDs with an altered dimension showed a random distribution of DEGs around them (Figure S3B).

**Figure 3:**
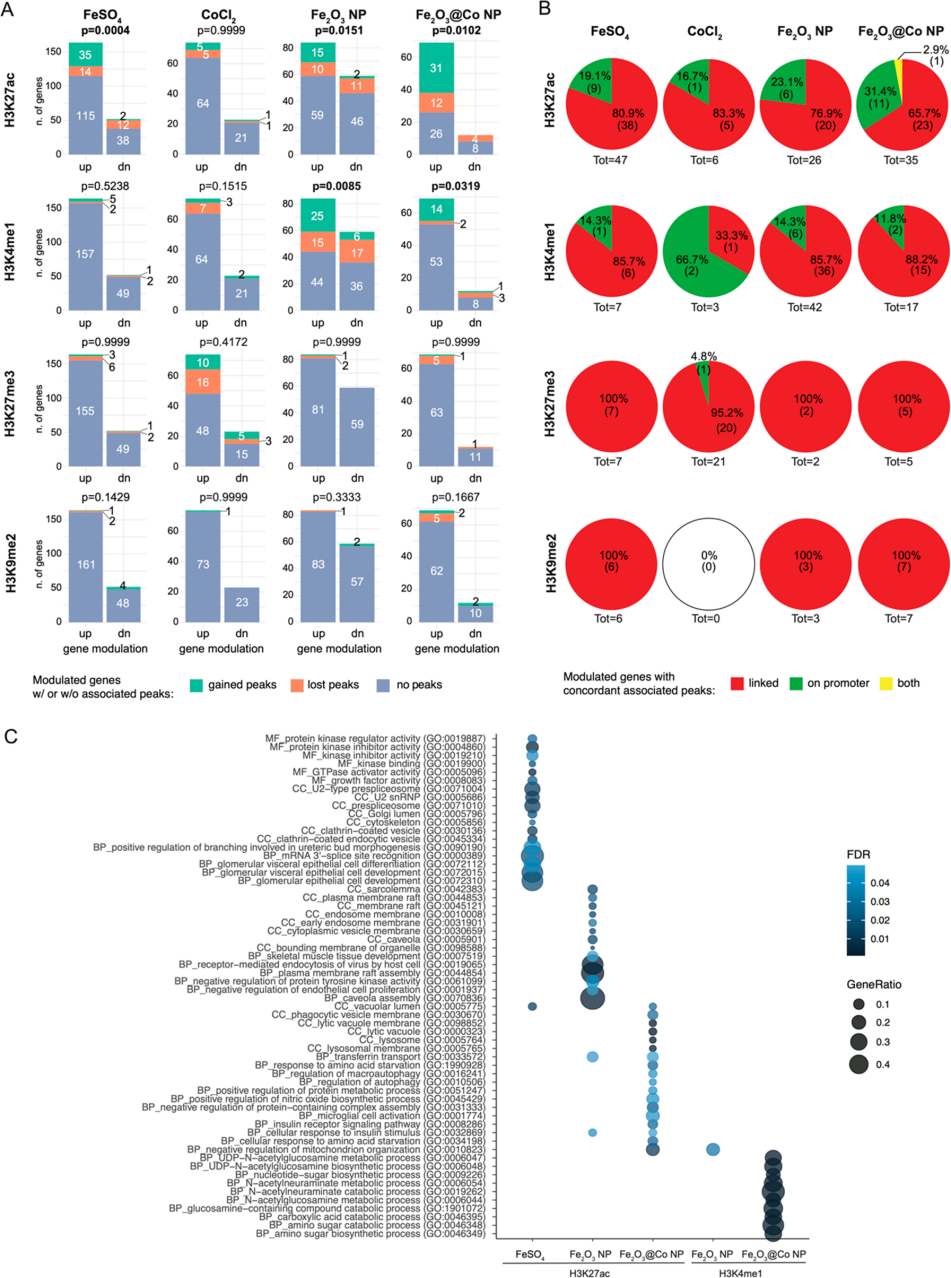
Association analysis between ChIP-seq and RNA-seq data sets. (A) Stacked bar charts of the association between differential peaks for each histone modification and the transcriptional modulation of genes associated with them for the four treatments. Fisher’s exact test was performed on modulated genes vs modulated peaks. (B) Pie charts of the percentage of differential peaks for the histone modifications mapping to either within promoter regions [i.e., regions located within 1000 base pairs of a transcription start site (TSS)] or outside of them for each treatment. (C) Gene ontology analysis for biological processes (BPs), molecular function (MF), and cellular component (CC) of genes regulated by H3K27ac and H3K4me1 in NIH3T3 cells exposed to 6.25 μg/mL NPs or FeSO_4_ for 48 h.

GO analysis has revealed that H3K27ac mediates the transcriptional effect of both NPs on genes linked to toxicity processes, such as cellular uptake (e.g., lysosome, lytic vacuole membrane, cytoplasmic membrane, and membrane raft) and metabolism. H3K27ac has also regulated the expression of genes involved in autophagy in cells exposed to Fe_2_O_3_@Co NPs, as well as of RNA-splicing genes and glomerular epithelial cell development in cells exposed to FeSO_4_ (Figure 3C). With regard to H3K4me1, redistribution was linked to dysregulation of genes involved in glucosamine metabolism in cells exposed to Fe_2_O_3_@Co NPs (Figure 3C).

The findings presented above indicate that the various histone marks play different roles in the gene expression changes caused by Fe_2_O_3_ NPs, Fe_2_O_3_@Co NPs, or FeSO_4_. H3K27ac had a major impact on transcription in cells exposed to both NPs and FeSO_4_, whereas H3K4me1 mediated changes only after NP treatment.

H3K27ac is a key regulator of promoters and enhancers.^25^ Thus, we wondered which of these regulatory elements mediated the transcriptional changes resulting from treatment with NPs and FeSO_4_. To address this question, we mapped H3K27ac DPs to genomic regions associated with one or more DEGs. To define the function of these genes, we performed functional annotation analysis. As shown in Figure 3B, we found that, in Fe_2_O_3_ NP-exposed cells, 76.9% of the redistribution of H3K27ac occurred outside promoter regions: this was 65.7% in cells exposed to Fe_2_O_3_@Co NPs and 80.9% in cells exposed to FeSO_4_. Thus, the two NPs and FeSO_4_ promote transcriptional changes preferentially through H3K27ac-mediated activation of enhancers with the activation of promoters playing a minor role. In support of these findings, the redistribution of H3K4me1, an enhancer-associated histone mark, occurred preferentially outside the promoter regions (Figure 3B).

Interestingly, 115 and 25 H3K27ac-regulated enhancers and promoters, respectively, were identified in at least one treatment condition (Figure 4A). These sets of regulatory elements were associated with 95 and 25 genes, respectively, including genes linked with toxicity such as metallothionein family genes (*Mt1* and *Mt2*), genes of vacuole lytic link disease (e.g., *AP3S1* and *ctsb*), cancer-associated genes (*Cav1*, *Cav2*, *Cblb*, and *TGFbI*), and genes involved in the formation of specialized membrane domains (*Ank*, *Ankrd13b*, and *Ankrd26*). We confirmed the transcriptional changes and H3K27ac and H3K4me1 redistribution observed previously through analysis of RNA-seq and ChIP-seq read distributions (Figure 4B).

**Figure 4:**
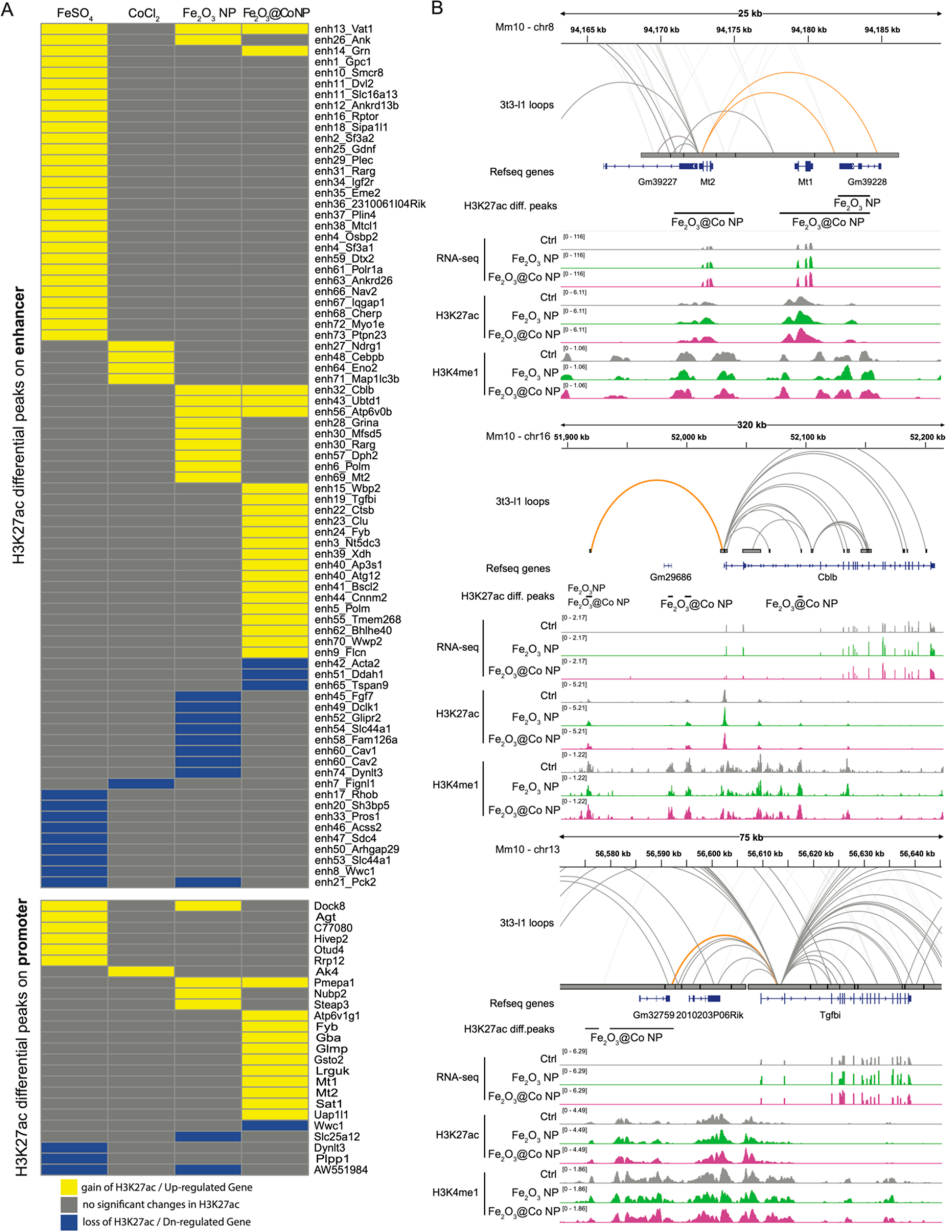
(A) Table of the activation status of 115 enhancers (top) and 25 promoters (bottom) found regulated by H3K27ac under at least one treatment condition. The activation status of regulatory regions was defined on the basis of H3K27ac redistribution. A gain in H3K27ac deposition was considered to indicate activation (yellow), whereas a loss of H3K27ac corresponded with repression (blue). (B) RNA-seq and ChIP-seq profiles for H3K27ac and H3K4me1 on metallothionein 1 (*Mt1*), metallothionein 2 (*Mt2*), cbl proto-oncogene B (*Cblb*), and transforming growth factor β-induced (*Tgfbi*) genes in NIH3T3 cells that were not exposed (negative control, ctrl) or exposed to either Fe_2_O_3_ NPs or Fe_2_O_3_@Co NPs. Arcs connecting enhancers with promoters are colored orange, whereas differential peaks for H3K27ac are indicated with a dark line.

In conclusion, we provide evidence of the role of H3K27ac and H3K4me1 in defining the transcription program regulating the nanotoxicity of Fe_2_O_3_ NPs and Fe_2_O_3_@Co NPs, two iron-based magnetic NPs, which were designed by the European Hotzyme consortium to remotely control enzyme activation. We also reveal that H3K27ac mediates the transcription effects of these NPs mainly through the regulation of enhancer activity. It is noteworthy that we found that each of the two NPs used regulates the activity of a distinct set of enhancers, and furthermore, these genetic elements are different from those found altered after treatment with FeSO_4_. Interestingly, for CoCl_2_, a component of Fe_2_O_3_@Co NPs, we did not observe an effect of the histone modifications analyzed on transcriptional changes. Given that the two NPs had a similar impact on cell viability at the time and concentration analyzed, we can exclude that their epigenetic effects reflect their different cytotoxicity. However, we cannot rule this out for FeSO_4_ and CoCl_2_.

These findings support two assumptions: (1) that the distinct transcription responses that NIH3T3 cells present after exposure to two different iron-based NPs are the result of distinct changes of the epigenetic landscape and (2) that enhancer regions are the genetic elements most involved in mediating the epigenetic effects related to nanotoxicity. Enhancers are *cis*-regulatory elements that define the transcription level of genes involved in several biological processes, including cell differentiation and in maintaining cell homeostasis.^26^ Alteration of their activity can compromise cell differentiation^27^ and can also lead to several diseases (e.g., cancer and heart failure).^28^ Therefore, we propose that the epigenetic impairment of the activity of these genetic elements via H3K27ac could mediate the toxic effects of NPs on living organisms. In support of this, we found that enhancer-dependent transcription modulation promotes the expression of genes linked with several diseases, including *Ctsb*, *Cav1*, and *TGFbI*. *Ctsb* encodes cathepsin B, a cysteine protease whose level of expression increases as a result of the duplication of enhancer regions causing keratolytic winter erythema.^29^ The level of expression of *Cav1*, which encodes caveolin 1-a, has been shown to increase in cancer, promoting tumor progression and metastasis.^30^ Finally, *TGFbI* encodes induced transforming growth factor β, which plays a key role in metastasis and drug resistance in cancer.^31^ Moreover, *TGFbI* is also involved corneal dystrophies.^32^ Metallothioneins (MTs) make up a family of cysteine-rich metal-binding proteins^33^ that are involved in metal homeostasis and, importantly, in the response to a wide range of stress conditions ranging from exposure to various molecules, metal NPs, to excessive animal crowding.^34,35^ Deregulation of the expression of these proteins has also been linked to carcinogenesis and cancer drug resistance. For these reasons, the expression of MTs was the subject of several studies. Although it has been shown that DNA methylation and histone deacetylation are involved in the repression of *Mt-1* in cancer cells,^36^ the existence of a unique epigenetic signature underlying their regulation is not clear. Our data contribute to clarifying this aspect, showing that the upregulation of *Mt-1* and *Mt-2* in response to exposure to metal NPs depends on an increase in the level of H3K27ac in the promoter regions of both genes and in the enhancer region coupled to MT-2.

Heterochromatin is a region of highly condensed chromatin that promotes transcriptional silencing.^37^ Impairment of this type of chromatin organization can lead to alterations of gene expression programs causing several diseases.^38^ Recent studies have also suggested a role of heterochromatin in mediating the effects of various stress stimuli.^39,40^ Although these findings render heterochromatin an enticing target for understanding the molecular mechanisms of nanotoxicity, we did not detect the involvement of H3K9me2 and H327me3, two key histone modifications regulating heterochromatin organization, in defining the changes in transcription caused by NPs and their corresponding ions (i.e., iron and cobalt). These data suggest that heterochromatin is not involved in defining the transcriptional changes caused by treatment with the two NPs. However, due to the dynamic nature of histone modifications, and the crosstalk between different epigenetic marks,^39^ we cannot exclude the possibility that H3K9me2 and H3K27me3 could act either before or after the time point that we analyzed (48 h), or the involvement of other repressive epigenetic mechanisms (e.g., DNA methylation, H3K9me3, and H4K20me3) in mediating the effect of the two NPs on heterochromatin.

It was reported that small doses after short periods of exposure of iron-based NPs can cause adverse effects.^41^ However, the mechanisms underlying this nanotoxicity are largely unknown. Indeed, although reactive oxygen species (ROS) were found to be an important mediator of toxicity for several nanomaterials,^42^ and given the fact that their production can be induced in cells after exposure to iron NPs via the Fenton reaction,^43^ the amount of free radicals produced by iron NPs is not enough to cause oxidative stress.^44^ Here, although we do not identify the specific molecular pathways by which iron NPs alter the epigenetic landscape, we show that changes in the genomic distribution of H3K27ac and H3K4me1 play a role in nanotoxicity. However, other epigenetic mechanisms not evaluated here could potentially play a role in mediating nanotoxicity.

It is noteworthy that among the genes that we found to be dysregulated by iron via H3K27ac-dependent modulation of enhancer activity, there were genes involved in neuronal diseases (e.g., *Vat*, *GDNF*, and *NAV2*) and cancer (e.g., *RARG* and *IGF2R*). Because the excessive absorption of iron can lead to cancer^45^ and neurodegenerative diseases such as Alzheimer’s and Parkinson’s disease,^46^ we hypothesize that the toxic effect of iron could be due to not only the production of free radicals^45,46^ but also changes in the epigenetic landscape that leads to dysregulation of genes involved in various diseases.

Moreover, because alterations to the epigenetic landscape can accumulate in the cell over time, suggesting a role of epigenetic regulation in latent toxicity,^47^ our data support this hypothesis and provide a set of genomic regions that could be useful for the development of assays that can assess this type of toxicity, an aspect that the currently available toxicological assays fail to measure.

It is worth noting that the epigenetic responses to environmental stimuli are not necessarily harmful. They can, indeed, be adaptive and cause hormesis,^48^ a phenomenon in which adaptive responses to small doses of otherwise harmful conditions improve the functional ability of cells and organisms.^49^ In this context, although the concentrations used are not “non-effect”, we can imagine that some of the observed epigenetic responses could have been also caused by much lower concentrations (“hormetic”).

A limitation of this study is that the very same NPs at the same dose and exposure time may induce different transcriptional responses in different cell lines.^35,8^ Furthermore, the gene expression time course is not the same for all of the genes, and consequently, the choice of the exposure time might drastically impact the results. In this regard, the selected epigenetically regulated genes should be re-evaluated as a function of time and tissue.

## Supporting Information Available

Lists of differential peaks for histone marks (XLSX)
Lists of differentially expressed genes (XLSX)
Materials and methods and Figures S1 and S3 (PDF)
