# Supplementary material for: Iron Oxide Nanoparticles with and without Cobalt Functionalization Provoke Changes in the Transcription Profile via Epigenetic Modulation of Enhancer Activity
Source: Nano Lett. 2023 Jul 26;23(19):9151–9. doi: 10.1021/acs.nanolett.3c01967 (PMC10571150; doi:10.1021/acs.nanolett.3c01967)
Supplement: Supplementary file 3 — nl3c01967_si_003.pdf [file nl3c01967_si_003.pdf]

Iron oxide nanoparticles with and without cobalt functionalization provoke changes in the transcription profile via epigenetic modulation of enhancer activity

Federica Gamberoni[a]†, Marina Borgese[b]†, Christina Pagiatakis[a,c], Ilaria Armenia[d], Valeria Grazù[d], Rosalba Gornati[a], Simone Serio[a,c,e]*, Roberto Papait[a,c]*, Giovanni Bernardini[a]*

[a]Department of Biotechnology and Life Sciences, University of Insubria, via J.H. Dunant 3, 21100, Varese, Italy
[b]Department of Medicine and Surgery, University of Insubria, via Guicciardini 9, 21100 Varese, Italy
[c]IRCCS Humanitas Research Hospital, via Manzoni 56, 20089, Rozzano, Milan, Italy
[d]BioNanoSurf Group, Instituto de Nanociencia y Materiales de Aragón (INMA, CSIC- UNIZAR), Edificio I +D, 50018 Zaragoza, Spain
[e]Department of Biomedical Sciences, Humanitas University, via Rita Levi Montalcini 4, Pieve Emanuele, MI, Italy

†These authors contributed equally to this paper

*Corresponding authors e-mails: simone.serio@humanitasreserach.it, roberto.papait@uninsubria.it, giovanni.bernardini@uninsubria.it

Materials and methods

Cell culture

Mouse embryonic fibroblast NIH/3T3 cells (ATCC® CRL-1658), obtained from ATCC, were cultured in fresh complete medium, composed of Dulbecco's modified Eagle medium (DMEM) with high glucose supplemented with 10% fetal bovine serum, 1% L-glutamine and 1% penicillin/streptomycin solution, at 37°C in a humidified 5% CO_2 atmosphere and passaged when the cells reached around 70-80% of confluence.

CellTiter-Glo® Luminescent Cell Viability Assay

For cytotoxicity experiments, 3600 cells were seeded into a 96-well plate in a fresh medium and maintained for 24 h at 37°C in 5% CO_2 and allowed to adhere before the treatment. The cells were exposed to increasing concentrations of NPs, corresponding to 0.625, 1.25, 2.5, 5 and 10 µg in terms of iron (in 100 µl final volume) and their corresponding salts ($FeSO_4 \cdot 7H_2O$ and $CoCl_2 \cdot 6H_2O$) for 48 h. The cells cultured without NPs and ions were used as a control group. The range of concentration of NPs and salt was determined in preliminary experiments (not reported here). NP suspensions were prepared fresh before each treatment in culture medium. Cell viability was determined measuring ATP content by CellTiter-Glo® Luminescent Cell Viability Assay (Promega, Milan, Italy) according to the manufacturer's instructions. After 48 h of treatment, multi-well plates were equilibrated for 30 min at room temperature before adding a volume of CellTiter-Glo reagent equal to the volume of cell culture medium present in each well. Plates were shaken for 2 min to induce cell lysis and then, left at room temperature for 10 min to stabilize the luminescent signal. The signal was recorded using the Glomax Discover plate reader (Promega, Milan, Italy).

ChIP assay

Genome-wide localization of histone modifications (H3K27Ac, H3K27me3, H3K4me1 and H3K9me2) was determined by Chromatin immunoprecipitation followed by high-throughput sequencing. The procedure was performed as described elsewhere [1].

Briefly, cells for immunoprecipitation were treated as described above. After 48 h of treatment, cells were cross-linked for 10 minutes at room temperature with 1% formaldehyde. The reaction was quenched by adding 0.125 M glycine. After several washes in PBS to remove the glycine, cells were collected by centrifugation and then, resuspended in lysis buffer (5 mM PIPES pH 8, 85 mM KCl, 0.5% NP40, and protease inhibitors). Cells were incubated on ice for 30 minutes before proceeding with sonication to produce chromatin fragments ranging from 200 to 400 bp. The efficiency of sonication was confirmed with agarose gel electrophoresis. The samples were pre-cleared for 1 hour with protein-G agarose beads and then immunoprecipitated overnight at 4°C with the following antibodies: anti-H3 (Abcam, ab1791), rabbit IgG (Millipore, 12-370), anti-H3K27me3 (Millipore, 07-449), anti-H3K9me2 (Abcam, ab1220) and anti-H3K27ac (Abcam, ab4729) anti-H3K4me1 (Abcam, ab8895). After incubation, the immunocomplexes were bound to protein-G beads for 2 hours and then washed 5 times with low-salt wash buffer (0.1% SDS, 2 mM EDTA, 20 mM Tris HCl pH 8, 1% Triton X-100, 150 mM NaCl, and protease inhibitors), 4 times with high-salt wash buffer (0.1% SDS, 2 mM EDTA, 20 mM Tris HCl pH 8, 1% Triton X-100, 500 mM NaCl, and protease inhibitors), and finally with TE buffer. Immunocomplexes were eluted in elution buffer (1% SDS, 100mM NaHCO3, and protease inhibitors), and de-cross-linked at 65°C overnight. Samples were treated with proteinase K, extracted with phenol/chloroform, and precipitated with ethanol. Purified DNA was sequenced by QuantiFluor ONE dsDNA System (Promega, Milan, Italy). Next generation sequencing was performed by IGA Technology services, Udine (Italy).

ChIP sequencing and analysis

Two biological replicates of NIH3T3 cells exposed to Fe_2O_3 NPs, Fe_2O_3@Co NPs, $FeSO_4$, or $CoCl_2$ were profiled for each histone modification (H3K27Ac, H3K27me3, H3K4me1 and H3K9me2). The libraries were generated using Ovation® Ultralow V2 DNA-Seq Library Preparation Kit (Tecan/NuGEN, Redwood City, CA). ChIP-seq libraries were obtained from approximately 2 ng of double-stranded DNA. The workflow consisted of three steps: end repair to generate blunt ends, adaptor ligation with optional multiplexing, and PCR. Paired-end sequence reads (150 bp in length) were generated on a NovaSeq6000 instrument (Illumina, San Diego, US). We obtained 88±26 million reads per sample.

ChIP-seq libraries were processed using the ENCODE Transcription Factor and Histone ChIP-Seq pipeline (https://github.com/ENCODE-DCC/chip-seq-pipeline2; v2.1.4). Briefly, fastq files were aligned with Bowtie2 v2.3.4 [2] and then filtered to remove duplicates with Picard v2.20.7[3], multi-mapping reads and low-quality alignments with samtools v1.9[4]. MACS2 v2.2.4[5]was used to call peaks from the filtered alignments using p-value < 0.05. In addition to calling peaks on replicates, peaks were called on pooled data and pooled pseudo-replicates obtained from the pooled set by splitting alignments into two equal sets. Peaks in the ENCODE mm10 blacklist were filtered out. The filtered peak sets were then assessed for reproducibility using IDR v2.0.4.2[6]. The final peak set used was the "optimal" peak set from the pipeline with IDR cut-off of 0.05 (Zenodo).

Differential peak calling of ChIP-seq replicates was performed with csaw v1.24.3[7] and voom function from limma v.3.46[8] package starting from MACS2 final peaks identified in at least one of the five conditions for the same antibody. Differential peaks (DPs) were filtered by FDR adjusted p-value < 0.1 and |log2FC| > 0.3. Deeptools[9] was used to analyze the correlation between ChIP-seq samples. Gene ontology analysis was performed using enrichR[10] package in R on DP nearby genes (the two closest genes) identified using closestBed function of BEDtools[11] within 250kb. For each condition, we selected the top ten GO terms that were statistically significant for the fisher exact test (FDR-adjusted p-value < 0.05). Hierarchical clustering of the scores, the negative log10(FDR-adjusted p-value), for the union of all terms over all the conditions was performed using hclust and dist functions in R. Clustering was performed with the ward.D method and Pearson's correlation distance to generate a heatmap using pheatmap[12] package in R without scaling. The similarities between ChIP-seq libraries for the same antibody were examined through the multi-dimensional scaling (MDS) plots using the plotMDS() function from edgeR[13] package in R.

RNA extraction

Total RNA was isolated from NIH-3T3 cells after 48 hours of treatment. RNA was purified using the Direct-zol RNA MiniPrep kit (Zymo Reserch) according to the manufacturer's protocol. The integrity of extracted RNA was evaluated with agarose gel electrophoresis. Total RNA amount was quantified by QuantiFluor RNA System (Promega, Milan, Italy) and finally the analysis of next generation sequencing was performed by IGA Technology services, Udine (Italy).

RNA sequencing and analysis

Three biological replicates of NIH3T3 cells treated with Fe_2O_3 NPs, Fe_2O_3@Co NPs, FeSO4, or $CoCl_2$ were profiled. RNA-seq library preparation was performed with Universal Plus mRNA-Seq kit (Tecan Genomics, Redwood City, CA) following the manufacturer's instructions (library type: fr-secondstrand). Paired-end multiplexed libraries were sequenced using the NovaSeq 6000 instrument (Illumina, San Diego, US). 146−188 million reads/sample were obtained. Paired-end reads of 150bp were aligned to the GENCODE Mus musculus reference genome (build GRCm38/mm10) using STAR v2.7.2b[14]. Raw read counts were normalized with TMM implemented in edgeR[13] and lesser expressed genes were filtered out with the filterByExpr (min.count=40) function; differential expression analysis of read counts was performed using voom, lmFit and eBayes (robust=T) function of limma v.3.46[8] package in R. Significant differential genes were chosen based on an FDR < 0.1.

Sva v3.38[15] package was used to estimate artifacts (n.sv=1) and correct the CPM values. Hierarchical clustering of significantly modulated genes was performed using hclust and dist R functions on sva corrected log2CPM. Clustering was performed with the ward.D method and Pearson's correlation distance to generate a heatmap using pheatmap[12] and scaling the rows. Hierarchical clustering on principal components was performed using HCPC function of FactoMineR v2.7[16] package in R. The Venn Diagrams were obtained using the R package VennDiagram[17].

Ingenuity Pathway Analysis

QIAGEN IPA[18] (QIAGEN Inc., https://digitalinsights.qiagen.com/IPA) were run for four DEGs datasets: Fe_2O_3 NPs, Fe_2O_3@Co NPs, FeSO4, or CoCl2, versus control group. A comparative analysis was performed, and canonical pathways were filtered for enriched terms (p-value < 0.05 and $|z\text{-score}| \geq 1$). IPA z-scores with p-value > 0.05 were set to 0. The IPA z-score table was clustered with the ward.D method and Pearson's correlation distance to generate a heatmap using pheatmap[12] without scaling. Pathway terms with $|z\text{-score}| > 2$ were considered significant.

PCHi-C data analysis

The FASTQ files for Mouse 3T3-L1 pre-adipocyte PCHi-C data were downloaded from the GEO database: GSE95533. Raw read files were analyzed with HiC-Pro[19] pipeline using default parameters for HindIII digested DNA. Contact matrices were normalized with the ICE procedure[20]. Significant interactions detected by CHiCAGO[21] (score ≥5) were used for the downstream analysis.

Modulated *cis* regulatory elements in the four treatment conditions

The csaw[7] DPs were used to identify modulated regulatory regions physically interacting with promoters. PairToBed function of BEDtools[11] was used to find the modulated regulatory regions associated with modulated genes connected by looping interactions identified with PCHi-C data. Only interactions in accordance with epigenetic theory were selected. Fisher exact test was used to determine if there are non-random associations between selected modulated regulatory regions and linked modulated genes in the four treatment. Ggplots[22] package was used to plot the GO enrichment analysis, performed with enrichR[10], of fisher's significative (p-value < 0.05) gene sets.

K9-dimethyl domain, or H3K9me2-associated domain (KDD) identification

H3K9me2 domains were called using Enriched Domain Detector (EDD)[23] using the parameters '--fdr 0.05 -g 5 --bin-size 10'. Domains were compared for similarity using the Jaccard statistic as implemented by BEDtools[11]. The lengthening or shortening of KDDs on at least one side for the four exposure settings was calculated with bedops[24] using the parameter '–difference' and intersect function of BEDtools[11]. A custom perl script was used to find the log2 fold change (y axis) of genes localized +/- 200 KB around KDDs that underwent lengthening or shortening on at least one side for the four treatment conditions vs. the non-treated control. These data were plotted with Ggplots[22] package.

Data availability

RNA-seq and ChIP-seq data supporting the findings of this study are avaible from the NCBI BioProject database (https://www.ncbi.nlm.nih.gov/bioproject/) under accession code PRJNA971387.

Bibliography

(1) Papait, R.; Cattaneo, P.; Kunderfranco, P.; Greco, C.; Carullo, P.; Guffanti, A.; Vigano, V.; Stirparo, G. G.; Latronico, M. V.; Hasenfuss, G.; et al. Genome-wide analysis of histone marks identifying an epigenetic signature of promoters and enhancers underlying cardiac hypertrophy. *Proc Natl Acad Sci U S A* **2013**, *110* (50), 20164-20169. DOI: 10.1073/pnas.1315155110.
(2) Langmead, B.; Salzberg, S. L. Fast gapped-read alignment with Bowtie 2. *Nat Methods* **2012**, *9* (4), 357-359. DOI: 10.1038/nmeth.1923 From NLM Medline.
(3) Picard toolkit. Broad Institute: 2019.
(4) Li, H.; Handsaker, B.; Wysoker, A.; Fennell, T.; Ruan, J.; Homer, N.; Marth, G.; Abecasis, G.; Durbin, R.; Genome Project Data Processing, S. The Sequence Alignment/Map format and SAMtools. *Bioinformatics* **2009**, *25* (16), 2078-2079. DOI: 10.1093/bioinformatics/btp352.
(5) Zhang, Y.; Liu, T.; Meyer, C. A.; Eeckhoute, J.; Johnson, D. S.; Bernstein, B. E.; Nusbaum, C.; Myers, R. M.; Brown, M.; Li, W.; et al. Model-based analysis of ChIP-Seq (MACS). *Genome Biol* **2008**, *9* (9), R137. DOI: 10.1186/gb-2008-9-9-r137.
(6) Li, Q.; Brown, J. B.; Huang, H.; Bickel, P. J. Measuring reproducibility of high-throughput experiments. *The Annals of Applied Statistics* **2011**, *5* (3), 1752-1779, 1728.
(7) Lun, A. T.; Smyth, G. K. csaw: a Bioconductor package for differential binding analysis of ChIP-seq data using sliding windows. *Nucleic Acids Res* **2016**, *44* (5), e45. DOI: 10.1093/nar/gkv1191 From NLM Medline.
(8) Ritchie, M. E.; Phipson, B.; Wu, D.; Hu, Y.; Law, C. W.; Shi, W.; Smyth, G. K. limma powers differential expression analyses for RNA-sequencing and microarray studies. *Nucleic Acids Res* **2015**, *43* (7), e47. DOI: 10.1093/nar/gkv007 From NLM Medline.
(9) Ramirez, F.; Dundar, F.; Diehl, S.; Gruning, B. A.; Manke, T. deepTools: a flexible platform for exploring deep-sequencing data. *Nucleic Acids Res* **2014**, *42* (Web Server issue), W187-191. DOI: 10.1093/nar/gku365.
(10) Kuleshov, M. V.; Jones, M. R.; Rouillard, A. D.; Fernandez, N. F.; Duan, Q.; Wang, Z.; Koplev, S.; Jenkins, S. L.; Jagodnik, K. M.; Lachmann, A.; et al. Enrichr: a comprehensive gene set enrichment analysis web server 2016 update. *Nucleic Acids Res* **2016**, *44* (W1), W90-97. DOI: 10.1093/nar/gkw377.
(11) Quinlan, A. R.; Hall, I. M. BEDTools: a flexible suite of utilities for comparing genomic features. *Bioinformatics* **2010**, *26* (6), 841-842. DOI: 10.1093/bioinformatics/btq033.
(12) Raivo, K. pheatmap: Pretty Heatmaps. 2019.
(13) Robinson, M. D.; McCarthy, D. J.; Smyth, G. K. edgeR: a Bioconductor package for differential expression analysis of digital gene expression data. *Bioinformatics* **2010**, *26* (1), 139-140. DOI: 10.1093/bioinformatics/btp616.
(14) Dobin, A.; Davis, C. A.; Schlesinger, F.; Drenkow, J.; Zaleski, C.; Jha, S.; Batut, P.; Chaisson, M.; Gingeras, T. R. STAR: ultrafast universal RNA-seq aligner. *Bioinformatics* **2013**, *29* (1), 15-21. DOI: 10.1093/bioinformatics/bts635.
(15) Leek, J. T.; Johnson, W. E.; Parker, H. S.; Jaffe, A. E.; Storey, J. D. The sva package for removing batch effects and other unwanted variation in high-throughput experiments. *Bioinformatics* **2012**, *28* (6), 882-883. DOI: 10.1093/bioinformatics/bts034 From NLM Medline.
(16) Lê, S.; Josse, J.; Husson, F. FactoMineR: An R Package for Multivariate Analysis. *Journal of Statistical Software* **2008**, *25* (1), 1 - 18. DOI: 10.18637/jss.v025.i01 (acccessed 2023/03/08).
(17) Hanbo, C. VennDiagram: Generate High-Resolution Venn and Euler Plots. 2018.
(18) Kramer, A.; Green, J.; Pollard, J., Jr.; Tugendreich, S. Causal analysis approaches in Ingenuity Pathway Analysis. *Bioinformatics* **2014**, *30* (4), 523-530. DOI: 10.1093/bioinformatics/btt703 From NLM Medline.
(19) Servant, N.; Varoquaux, N.; Lajoie, B. R.; Viara, E.; Chen, C. J.; Vert, J. P.; Heard, E.; Dekker, J.; Barillot, E. HiC-Pro: an optimized and flexible pipeline for Hi-C data processing. *Genome Biol* **2015**, *16*. DOI: ARTN 259

10.1186/s13059-015-0831-x.
(20) Imakaev, M.; Fudenberg, G.; McCord, R. P.; Naumova, N.; Goloborodko, A.; Lajoie, B. R.; Dekker, J.; Mirny, L. A. Iterative correction of Hi-C data reveals hallmarks of chromosome organization. *Nat Methods* **2012**, *9* (10), 999-+. DOI: 10.1038/Nmeth.2148.
(21) Cairns, J.; Freire-Pritchett, P.; Wingett, S. W.; Varnai, C.; Dimond, A.; Plagnol, V.; Zerbino, D.; Schoenfelder, S.; Javierre, B. M.; Osborne, C.; et al. CHiCAGO: robust detection of DNA looping interactions in Capture Hi-C data. *Genome Biol* **2016**, *17* (1), 127. DOI: 10.1186/s13059-016-0992-2 From NLM Medline.
(22) Hadley, W. ggplot2: Elegant Graphics for Data Analysis. 2016.
(23) Lund, E.; Oldenburg, A. R.; Collas, P. Enriched domain detector: a program for detection of wide genomic enrichment domains robust against local variations. *Nucleic Acids Res* **2014**, *42* (11), e92. DOI: 10.1093/nar/gku324 From NLM Medline.
(24) Neph, S.; Kuehn, M. S.; Reynolds, A. P.; Haugen, E.; Thurman, R. E.; Johnson, A. K.; Rynes, E.; Maurano, M. T.; Vierstra, J.; Thomas, S.; et al. BEDOPS: high-performance genomic feature operations. *Bioinformatics* **2012**, *28* (14), 1919-1920. DOI: 10.1093/bioinformatics/bts277 From NLM Medline.
Figure 1

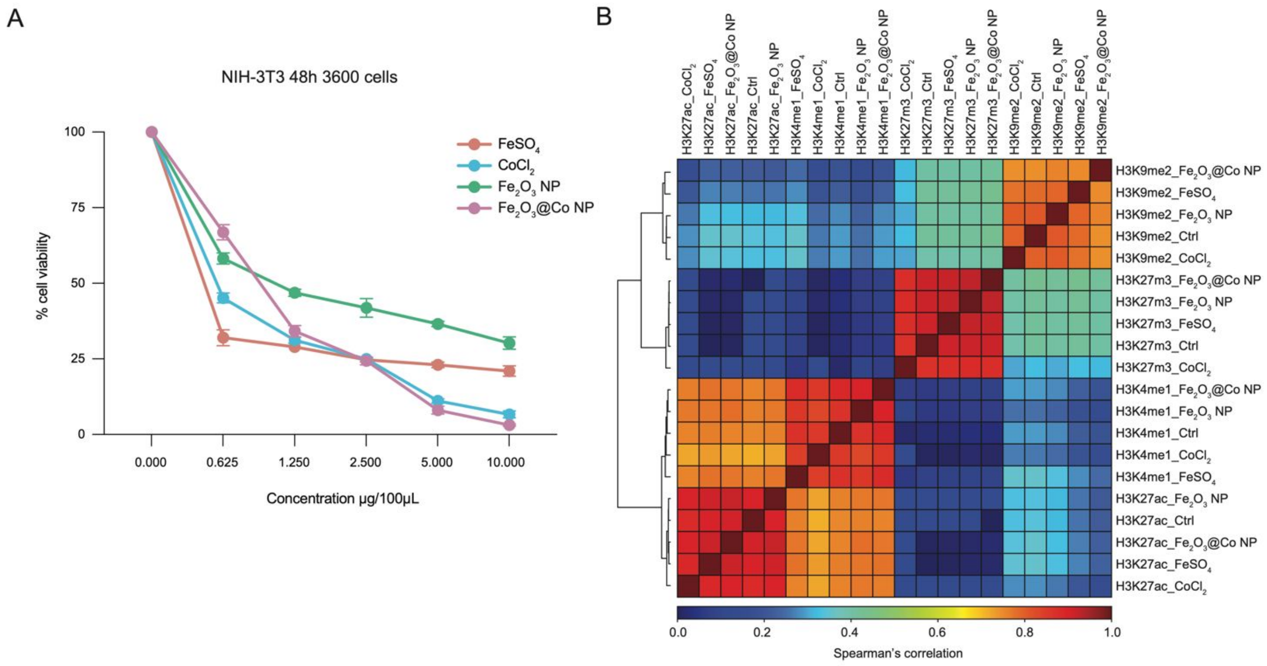

C

Comparison	CSAW differential peaks (FDR<0.1 & |log2FC|>0.3)		
	Gain peaks	Loss peaks	Total peaks
H3K27ac - $FeSO_4$ vs ctrl	1348	1349	2697
H3K27ac - $CoCl_2$ vs ctrl	917	853	1770
H3K27ac - Fe_2O_3 NP vs ctrl	1060	972	2032
H3K27ac - Fe_2O_3@Co NP vs ctrl	3249	3207	6456
H3K4me1 - $FeSO_4$ vs ctrl	123	164	287
H3K4me1 - $CoCl_2$ vs ctrl	613	679	1292
H3K4me1 - Fe_2O_3 NP vs ctrl	1804	2148	3952
H3K4me1 - Fe_2O_3@Co NP vs ctrl	599	606	1205
H3K27me3 - $FeSO_4$ vs ctrl	593	608	1201
H3K27me3 - $CoCl_2$ vs ctrl	7834	8013	15847
H3K27me3 - Fe_2O_3 NP vs ctrl	259	257	516
H3K27me3 - Fe_2O_3@Co NP vs ctrl	511	524	1035
H3K9me2 - $FeSO_4$ vs ctrl	349	323	672
H3K9me2 - $CoCl_2$ vs ctrl	218	210	428
H3K9me2 - Fe_2O_3 NP vs ctrl	59	72	131
H3K9me2 - Fe_2O_3@Co NP vs ctrl	753	789	1542

Figure S1. A) Graph of cell viability in NIH3T3 cells exposed to different concentrations (6.25, 12.5, 25, 50, and 100 µg/ml) of Fe_2O_3 NPs (blue line), Fe_2O_3@CoNPs (red line), $FeSO_4$ (yellow line), or $CoCl_2$ (light blue line) for 48h. Cell vitality was calculated against the control treatment (negative control, CTRL). B) Clustered heat map of pair-wise Spearman correlation of the genomic distribution of H3K27ac, H3K4me1, H3K9me2, and H3K27me3 in NIH3T3 cells exposed to NPs or ions. C) Table of the number of significant differential peaks identified for each histone modification (H3K27ac, H3K4me1, H3K9me2, and H3K27me3) for each of the four exposure settings. DPs with a false discovery rate (FDR) < 0.1 and |log2 fold change| > 0.3 were considered significant.

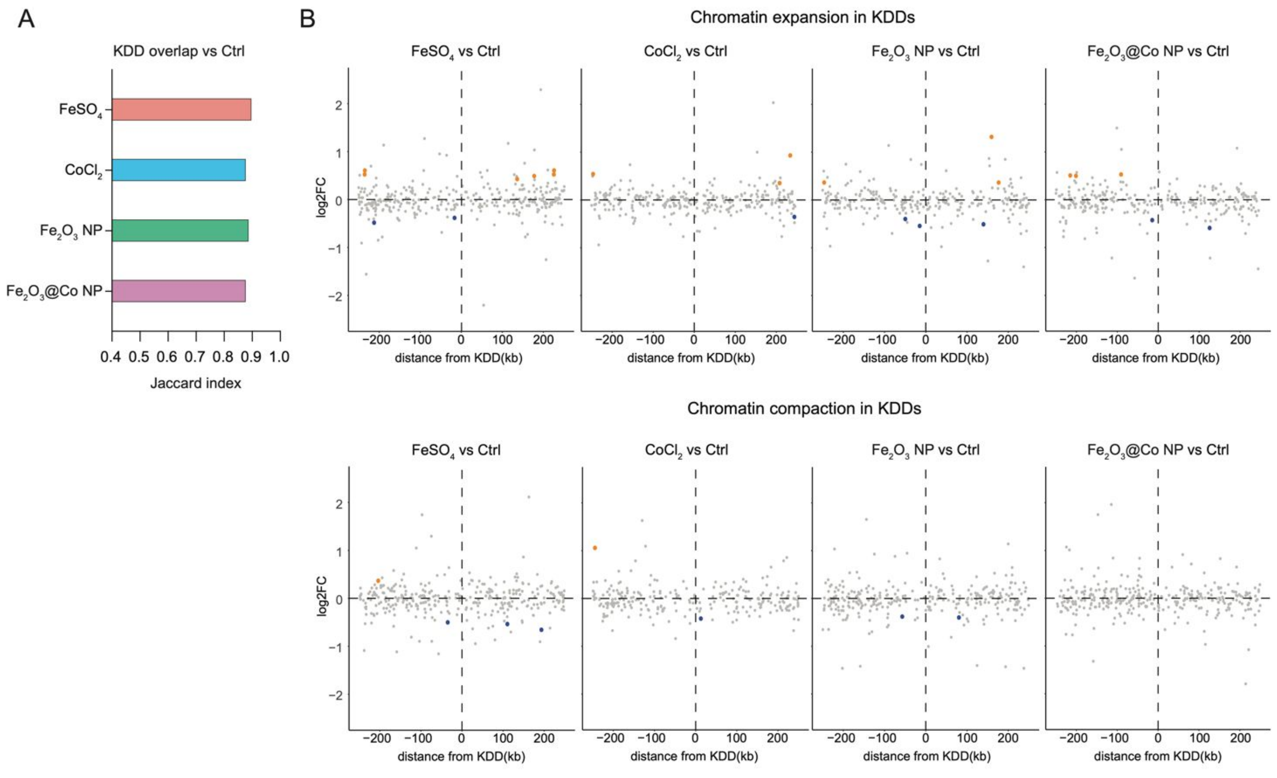

Figure S3: A) Bar plot of Jaccard similarity index calculated through the comparison of the genomic dimension of K9-dimethyl domains (KDD) in each treatment condition vs. the non-treated control. B) Graphs show the change of gene expression measured as log_2 fold change (y axis) of genes localized +/- 200 KB around KDDs (x axis) that underwent lengthening (upper panel) or shortening (lower panel) on at least one side for the four treatments. Yellow and blue dots indicate the DEGs that were up- and down-regulated, respectively.

Dataset S1: list of genomic regions with different enrichment for H3K27ac, H3K4me1, H3K9me2 and H3K27me3 in NIH3T3 cells exposed to 6.25 µg/ml of Fe_2O_3 NPs, Fe_2O_3@Co NPs, $FeSO_4$, or $CoCl_2$ for 48h, as resulting from ChIP-seq analyses.

Dataset S2: list of differentials expressed genes in NIH3T3 cells exposed to 6.25 µg/ml of Fe_2O_3 NPs, Fe_2O_3@Co NPs, $FeSO_4$, or $CoCl_2$ for 48h, as resulting from RNA-seq analyses.
